# ‘Blindness out of the womb’, a historical account of the first report of posterior reversible encephalopathy syndrome (PRES): choose your title well or your findings will be neglected

**DOI:** 10.1111/1471-0528.16004

**Published:** 2019-11-22

**Authors:** Wessel Ganzevoort, Kees Boterbloem, Joris van der Post, Otto Bleker

**Affiliations:** ^1^ Department of Obstetrics and Gynecology Amsterdam UMC University of Amsterdam Amsterdam the Netherlands; ^2^ USF History Department University of South Florida Tampa Florida USA

Dr Nicolaes Pieterszoon Tulp (1593–1674), appointed ‘Praelector Anatomiae’ (teacher of anatomy) at the Amsterdam Guild of Surgeons in 1629, is today famous from his depiction in ‘Anatomy Lesson’, painted by Rembrandt in 1632 (Figure 1). At the end of his life, he wrote a translation of the third Latin edition (1672) of his *Observationum Medicarum* (Medical Observations) into Dutch, maybe meant for his grandchildren. This unique manuscript is cared for in the archives of the House of Six in Amsterdam (Dudok van Heel et al. *Nicolaes Tulp: The Life and Work of an Amsterdam Physician and Magistrate in the 17th Century*, 2nd edn. Amsterdam: Six Art Promotion BV; 1998); a Dutch transcription was published in a limited edition in 1990. This recently came to our attention, and we realised that it contained the first description of posterior reversible encephalopathy syndrome (PRES), in chapter X of Book I, entitled ‘Blindness out of the womb’ (Apeldoorn & Beijer. *A complete facsimile and transcription of Tulp’s manuscript and a transcription, ‘Geneesinzichten van Dr. Nicolaes Tulp’*. Amsterdam: Six Art Promotion BV; 1990).

**Figure 1:**
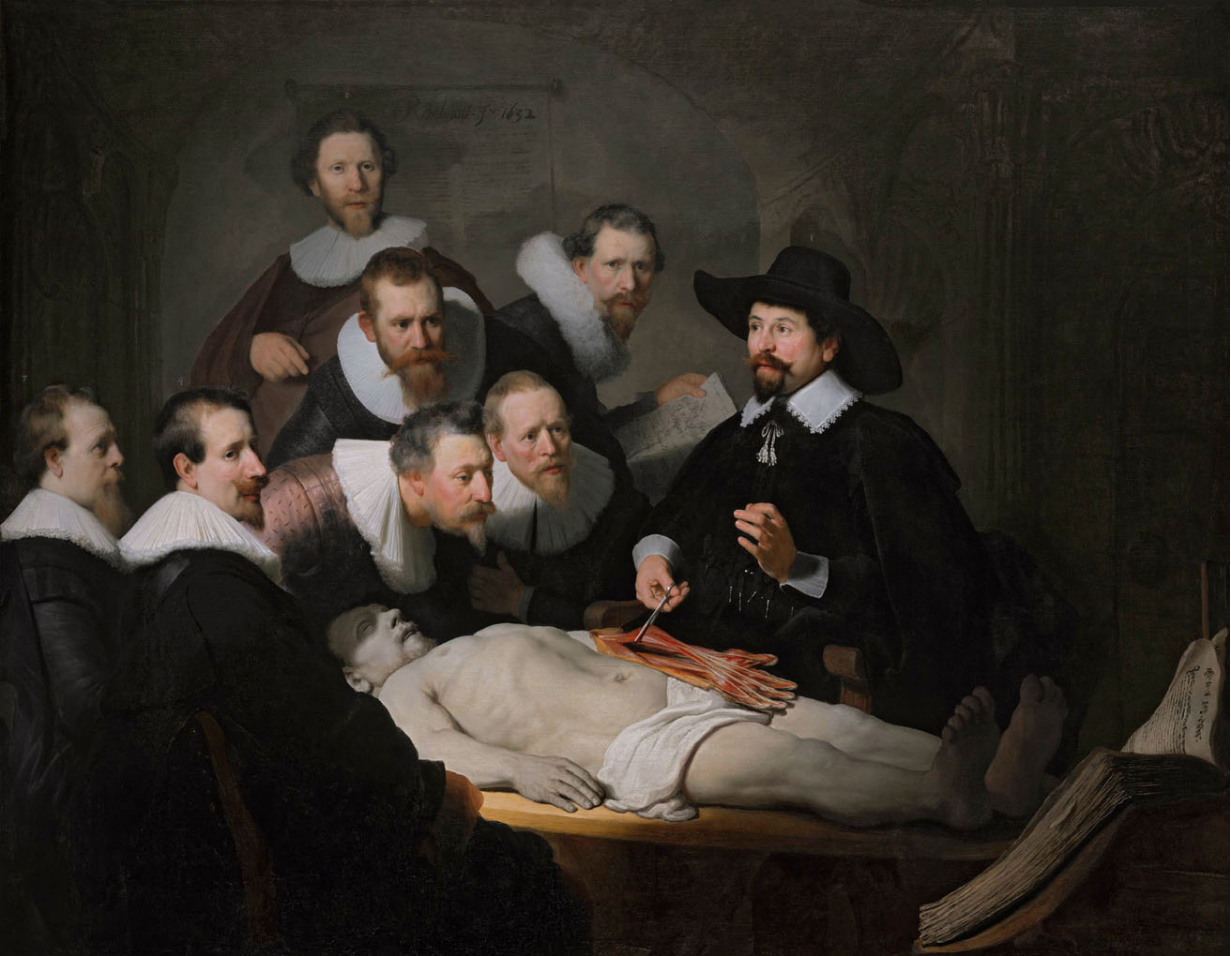
Rembrandt van Rijn *The Anatomy Lesson of Dr Nicolaes Tulp*, 1632. Canvas, 169.5 × 216.5 cm. Mauritshuis, The Hague, the Netherlands; inv. no. 146, available in the public domain.

Tulp described two cases of convulsions and unconsciousness during labour, of which the second ‘happened not long after [the first case] to our niece Maria Corver. However, she lost sight completely without any abnormalities of the black [pupil] of the eye or its external membranes, which kind of blindness the Ancients called darkness, while some recent authors named it gutta serena [clear droplet, i.e. blindness without any external symptom]’. In line with the contemporary paradigm, Tulp related the convulsions and the blindness to a congestion of blood in the brain. He ‘applied all available means of the art [of medicine]’ to the two patients and treated them respectively with bloodletting and large cupping glasses on the legs.

Because Tulp mentioned the patient to be ‘my niece Maria Corver’, we identified her as Maria Overrijn van Schoterbosch (1599/1600–1658); she married Dirck Corver (1587–1633), and they had three children. They were both members of the Dutch Republic’s nascent patriciate in Amsterdam’s golden age. Cornelis van der Voort painted portraits of Maria (Figure 2) and Dirck in 1622, and they are also available for public viewing.

**Figure 2:**
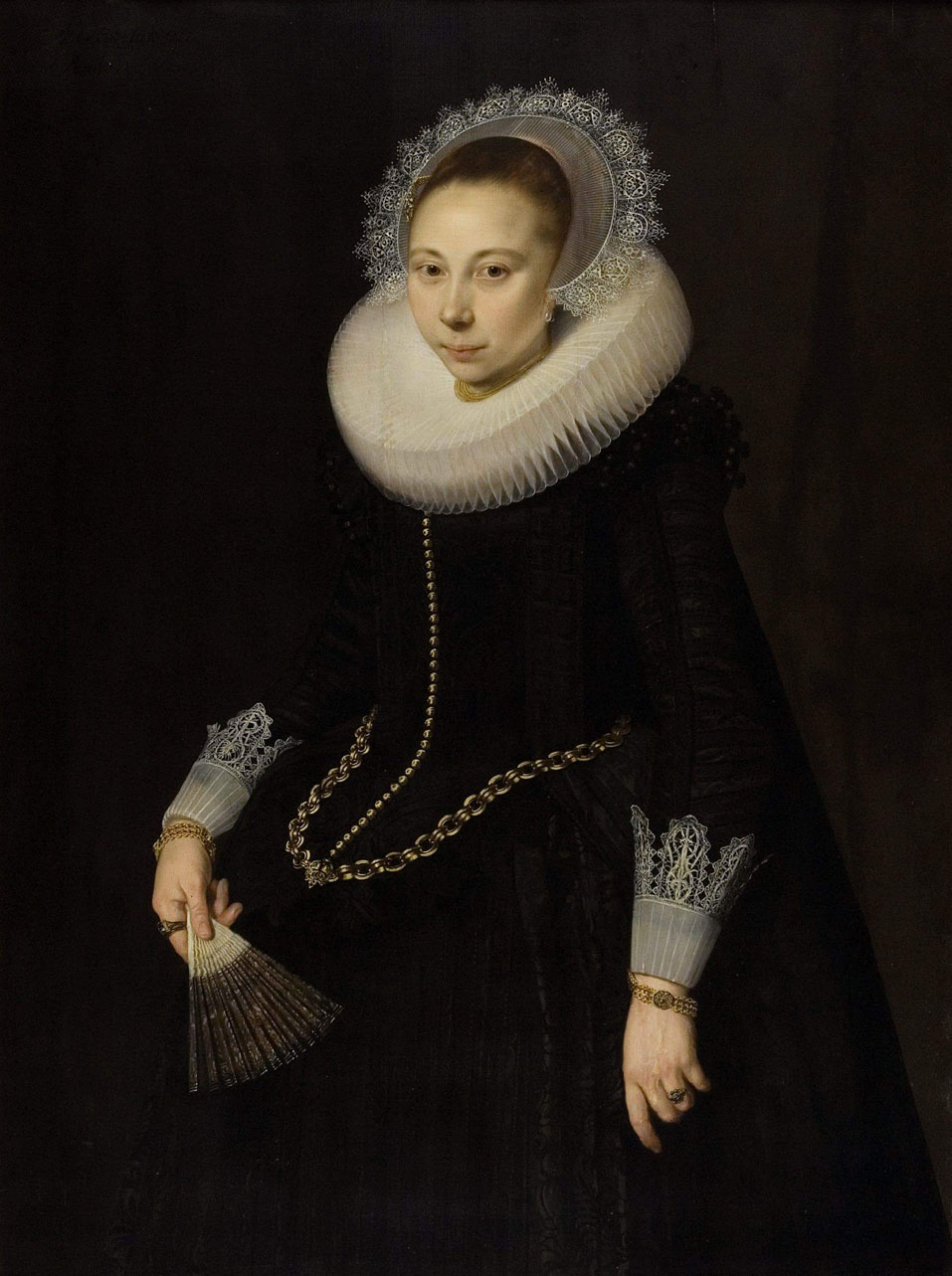
Cornelis van der Voort *Portrait of Maria Overrijn van Schoterbosch* (married to Dirck Corver), 1622. Panel, 90.6 × 122 cm. Rijksmuseum, Amsterdam, the Netherlands; inv. nr. SK‐A‐4765, available in the public domain.

In 1996, Hinchey et al. coined the term PRES for a pattern of headaches, vomiting, confusion, seizures, cortical blindness, and other visual abnormalities, accompanied by abrupt increases in blood pressure and impairment of renal function in patients who suffered from acute hypertensive encephalopathy associated with renal disease, received immunosuppressive therapy or interferon, or eclampsia (Hinchey et al. *N Engl J Med* 1996;334:494–500). Tulp’s excellent description of eclampsia‐related temporary blindness as the first published case report of PRES was probably overlooked because, in the three printed Latin editions of his medical observations (1641, 1652, and 1672), he titled chapter X of Book 1: ‘Convulsions out of the vulva’, not mentioning the blindness, nor the womb.

This story teaches us two things: first, this historical case report came about from careful observation, which is an important (but sometimes neglected) tool for clinicians, even in our time; second, this finding underpins the importance for every author – even Nicolaes Tulp – to choose the title of publications with care in order to reach the intended audience.

## Acknowledgements

We thank Jan Six X for his expertise and hospitality.

## Disclosure of interests

None declared. Completed disclosure of interests forms are available to view online as supporting information.
